# An Analytical Review of Different Approaches to Wastewater Discharge Standards with Particular Emphasis on Nutrients

**DOI:** 10.1007/s00267-020-01344-y

**Published:** 2020-08-12

**Authors:** Michał Preisner, Elena Neverova-Dziopak, Zbigniew Kowalewski

**Affiliations:** 1 Mineral and Energy Economy Research Institute, Polish Academy of Sciences, ul. Wybickiego 7A, Cracow, 31-261 Poland; 2 AGH University of Science and Technology, al. Mickiewicza 30, Cracow, 30-059 Poland

**Keywords:** Effluent quality standards, Eutrophication, Legal regulations, Wastewater discharge, Water policy

## Abstract

Despite the implementation of strict legal standards concerning nutrient loads within wastewater discharges in all European Union (EU) Member States it was not possible to achieve good ecological and chemical water status by 2015 in all EU countries. The main reasons for this situation are the imperfections of the legislation tools regarding the standardization of wastewater quality and the methodology of determining the conditions for wastewater introduction into receivers. The study aims to review and analyze the currently existing in various countries legal regulations setting the standards for wastewater discharged into receivers, which were intended for surface water protection and eutrophication mitigation. Besides the EU effluent standards, the regional and national regulations in chosen EU Member States (e.g., Germany, Sweden, and Denmark) have been reviewed. Moreover, the Helsinki Commission recommendations for signatory countries within the Baltic Sea catchment and the approaches for wastewater quality standardization in non-EU countries (e.g., Russia, Belarus, Switzerland, China, USA, Canada, and Dubai) were assessed. The analysis of the reviewed legal regulations allowed to diversify the methodological approaches for setting effluent quality standards in different regions and countries and to assess the effectiveness of existing legal tools in the field of eutrophication mitigation with the consideration of the environmental and economic reasonability. The results suggest that the receiver-oriented policies used among others in Switzerland and China are the most reasonable in terms of eutrophication mitigation.

## Introduction

Surface waters are the most important components of the environment and the necessary condition for human life (Schoumans et al. 2015). Therefore, water legislation in every country pays considerable attention to the legal regulations of their use and protection against pollution, ecological disturbance, illegal use, etc. Numerous legislative acts regulate the issues of water management by creating a licensing system, introducing several restrictions and prohibitions, legal liability measures, establishing the payments, penalties, etc. (Von Sperling and Augusto De Lemos Chernicharo 2002). Due to the fact that many water areas belong to several countries simultaneously they are also the subject to international regulations, in addition to national and regional laws. It makes the problem of their legal protection even more complicated because in some cases national law is not consistent with the requirements of numerous international conventions (Howarth and Marino 2006; Kupkanchanakul et al. 2015). The constant attention to the implementation of quality standards and their improvement is explained by the importance of water resources and their functions in the biosphere.

The first half of the 1970s can be considered as the beginning of targeted large-scale world activities in the standardization of the adverse environmental effects (Conley et al. 2009; Bohman 2018). Afterward, the formation of environmental management structures in developed countries began and water ecosystems were included in the priorities list (Risnik et al. 2012). Generally, the main idea of the current legislation on the regulation of discharges from point sources laid down in legal norms is to determine the maximum permissible concentrations/loads of pollutants in wastewater discharged into the receiver to ensure good water quality of the water body (Tsakiris 2015; Voulvoulis et al. 2017). According to the Water Framework Directive (2000/60/EC) classification of surface waters the “good ecological status” means a minimal deviation from undisturbed/natural state of water ecosystem under zero or minimal human influence, while the “good chemical status” is characterized by the undisturbed chemical state of waters. The “good” condition is defined descriptively in the Water Framework Directive, while normative values of water state indicators of different categories (biological, chemical, and hydromorphological) are set in the Water Law acts of various countries, which should correspond to good ecological and chemical status.

The discharge limit values (DLVs) established to reduce the nutrients loads introduced with effluents into surface waters in many countries are usually expressed as legally binding minimum standards. It may be expressed either as the pollutant concentrations in the effluent or as a discharged pollution load which may not be exceeded during a certain period. DLVs are usually applied regarding point sources where the effluents are discharged from wastewater treatment plants (WWTPs). Furthermore, DLVs should take into account local environmental conditions. Although such effluent standards do not reflect the actual response of the recipient and often are not justified enough from an ecological point of view (Inglezakis et al. 2016).

The Council Directive of May 21, 1991 (91/271/EEC; EC 1991) concerning urban wastewater treatment (UWWTD) seeks to reduce the pollution of freshwater, estuarial, and coastal waters contributed by wastewater and rainwater run-off (or their mixture) by introducing the restrictions on the discharged pollutant loads.

**Fig. 1:**
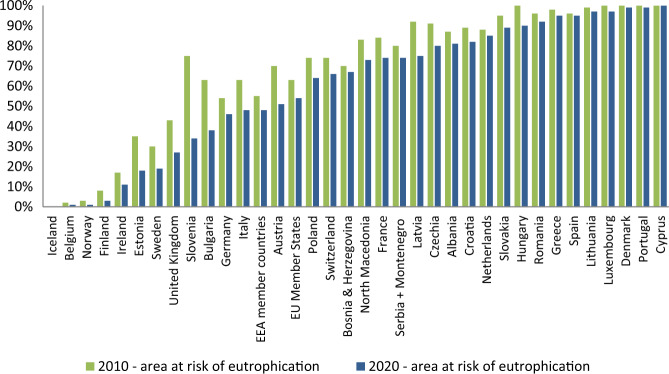
The ecosystem area at risk of eutrophication (based on: EEA 2020)

The inefficiency of 10 years of efforts undertaken by many European countries to prevent eutrophication caused by wastewater discharge based on the UWWTD guidelines is reflected by the share of water ecosystems at risk of eutrophication in 2010 and 2020 in Fig. 1. During the last 10 years of active implementation of that directive in European Union (EU) Member States the share of water ecosystems subjected to eutrophication still ranges from a few to over 90%, with an average share of such water areas. However, only a few countries have managed to achieve the measurable effect of reducing eutrophication risk, while such countries as Greece, Spain, Lithuania, Luxembourg, Denmark, Portugal, and Cyprus have over 90% of ecosystems area at risk of eutrophication (EEA 2020).

Recently, a serious discussion has begun about the revision of the environmental regulation system (Wang et al. 2017; Desmit et al. 2018). The standardization of the municipal wastewater quality discharged into water bodies is particularly carefully scrutinized. The economic situation of most water supply and wastewater enterprises and other water consumers depends on the level of financial expenditures they incur due to restrictive or inefficient standards that are not always justified (Smol et al. 2020). Moreover, the current effluent standards do not guarantee the ecological safety of aquatic ecosystems and bring to direct or indirect ecological damage and losses which is difficult to assess in monetary terms.

**Fig. 2:**
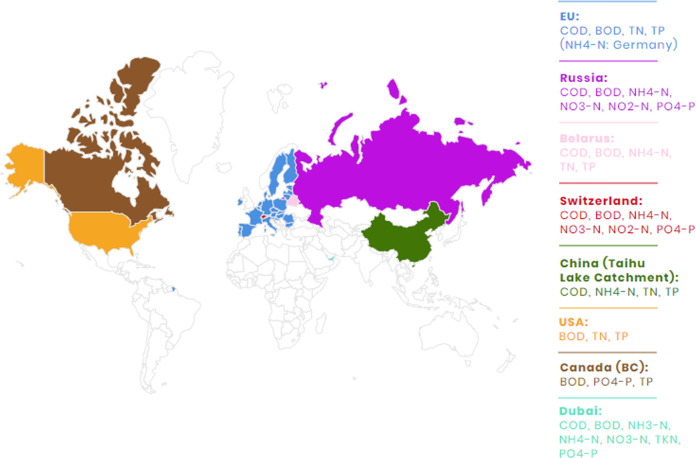
Studied geographical regions and obligatory wastewater quality parameters covered by legal regulations

The study aims to review and analyze the currently existing legal regulations in various countries for setting wastewater discharges standards. Figure 2 presents the studied areas and the pollution indicators included in legal regulations.

Legal regulations in chosen EU Member States (e.g., Germany, Sweden, and Denmark) and in non-EU countries (e.g., Russia, Belarus, Switzerland, China, USA, Canada, and Dubai) were carefully reviewed and assessed in terms of eutrophication mitigation effectiveness with special attention given to the environmental and economic reasonability.

## Methods

A comprehensive literature and legal acts review, analysis, and systematization were conducted to identify the existing wastewater discharge standards designed for water protection against pollution and eutrophication mitigation in chosen countries. The selection of primary references has been done based on the full-text databases (Elsevier Scopus, Elsevier ScienceDirect, SpringerLink, Google Scholar, and EUR-lex) and other available publications. The choice of literature was associated with the use of a few keywords: eutrophication, wastewater treatment, phosphorus removal, nitrogen removal, nutrients, legal regulations, effluent standards, discharge requirements, etc. Within the literature review, 69 references in English, Russian, and Polish were identified as relevant for the current study.

Based on the literature and legal acts review and analysis results, various methodological approaches for setting effluent quality standards in different regions and countries were identified. Within the analysis, the effectiveness of existing legal tools, used in various countries in the field of eutrophication mitigation, was assessed to show essential methodological differences in setting the effluent quality standards in the EU Member States and the non-EU countries from different regions of the globe.

### EU Regulations and their Implementation to the Member States Legislation

The main legal act regulating the quality of treated municipal wastewater discharged into receivers in EU countries is the UWWTD (so-called the Wastewater Directive). This Directive regulates the level of treatment by introducing required pollutants removal efficiency in treated wastewater discharged into receivers to protect aquatic ecosystems in the Member States.

The requirements were established for four WWTPs categories, depending on the size of the agglomeration, expressed by the population equivalent (PE), the type of wastewater receiver, and its sensitivity to eutrophication. Fifteen Member States: Austria, Belgium, Czech Republic, Denmark, Estonia, Latvia, Lithuania, Luxembourg, the Netherlands, Poland, Slovakia, Sweden, Finland, Bulgaria, and Romania have identified all their surface water bodies in their territory as sensitive areas (Zaragüeta and Acebes 2017). The next 13 countries: Croatia, Cyprus, France, Germany, Greece, Hungary, Ireland, Italy, Malta, Slovenia, Spain, Portugal, and United Kingdom considered only selected water areas as sensitive. After the accession to the EU, the Member States committed to the implementation of EU regulations, while the previous existing quality standards were reviewed and the legislation on the aquatic environment protection was amended.

An overview of the analyzed EU Member States legal requirements for regulating the conditions of wastewater introduction into receivers is presented in Table 1.

**Table 1:** The comparison of national legal regulations concerning treated wastewater quality parameters in selected EU Member States

Country/region	WWTP category	COD, mg/l	BOD_5_, mg/l	NH_4_^+^–N, mg/l	NO_2_^−^–N, NO_3_^−^–N, mg/l	TN, mg/l	PO_4_^3−^–P, mg/l	TP, mg/l	References
EU	<2000 PE	125	25	n/n^a^	n/n	n/n	n/n	n/n	EC (1991; 91/271/EEC)
	2000–10,000 PE	125	25	n/n	n/n	n/n	n/n	n/n	
	10,000–100,000 PE	125	25	n/n	n/n	15 (areas sensitive to eutrophication)	n/n	2 (areas sensitive to eutrophication)	
	<100,000 PE	125	25	n/n	n/n	10 (areas sensitive to eutrophication)	n/n	1 (areas sensitive to eutrophication)	
Germany	BOD_5_ < 60 kg/d (<1000 PE)	150	40	n/n	n/n	n/n	n/n	n/n	Federal Ministry of Environment Nature Conservation and Nuclear Safety (2002), BMU (2004)
	BOD_5_ < 300 kg/d (<5000 PE)	110	25	n/n	n/n	n/n	n/n	n/n	
	BOD_5_ < 1200 kg/d (<20,000 PE)	90	20	10	n/n	n/n	n/n	n/n	
	BOD_5_ < 6000 kg/d (<100,000 PE)	90	20	10	n/n	18	n/n	2	
	BOD_5_ < 6000 kg/d (>100,000 PE)	75	15	10	n/n	13	n/n	1	
Sweden	>2000 PE	n/n	15^b^ (BOD_7_)	n/n	n/n	15	n/n	0.5	Swedish (2016)
	2000–100,000 PE	n/n	15 (BOD_7_)	n/n	n/n	15	n/n	0.5	
	>100,000 PE	n/n	15 (BOD_7_)	n/n	n/n	10	n/n	0.5	
Denmark	n/n	75	10	n/n	n/n	8	n/n	0.4	Vind (2017)
HELCOM signatory countries	300–2000 PE	n/n	25	n/n	n/n	35	n/n	2	HELCOM (2007)
	2000–10,000 PE	n/n	15	n/n	n/n	30	n/n	1	
	10,000–100,000 PE	n/n	15	n/n	n/n	15	n/n	0.5	
	>100,000 PE	n/n	15	n/n	n/n	10 (8^c^)	n/n	0.5	

^a^*n/n* not normalized parameter

^b^1 mg/l BOD_7_ = ~0.83 mg/l BOD_5_

^c^According to the obligations of Sankt Petersburg

#### Requirements for treated wastewater quality in Germany

The most important national regulations in the field of wastewater management in Germany are, among others, the Ordinance on Requirements for the Discharge of Wastewater into Waters (AbwV), the Federal Water Act (WHG), and other binding EU regulations.

The AbwV establishes quality standards for five categories of WWTPs depending on the wastewater 5-day biochemical oxygen demand (BOD_5_) load (Appendix 1 to the Regulation): cat. 1—BOD_5_ < 60 kg/d (<1000 PE), cat. 2—BOD_5_ < 300 kg/d (<5000 PE), cat. 3—BOD_5_ < 1200 kg/d (<20,000 PE), cat. 4—BOD_5_ < 6000 kg/d (<100,000 PE), cat. 5—BOD_5_ < 6000 kg/d (>100,000 PE). The discharge requirements apply to ammonium nitrogen (NH_4_^+^–N) and total nitrogen (TN) if the wastewater temperature is above 12 °C regarding the effluent from the biological reactor of the WWTP. The 12 °C criterion may be replaced by an alternative seasonal restriction regarding the summer season—from May 1 to October 31.

German legislation provides the possibility for setting different national administrative regulations regarding the quality of treated wastewater, dependent not only on the WWTP capacity but also on the type and properties of the effluent receiver. In Bavaria and North Rhine-Westphalia, for example, the conditions for wastewater discharges into receivers are determined based on local administrative regulations (Henneberg and Triebskorn 2015). Stricter requirements are imposed on wastewater discharged into Lake Constance (*ger. Bodensee*) where the local limit values of pollutants in treated wastewater may differ from the German Wastewater Framework Regulation (AERZEN 2019). For example, the limit for total phosphorus (TP) in treated wastewater discharged into Lake Constance is 0.3 mg/l (Wilson et al. 2019). In this way, Germany reserves the right to establish regional effluent standards, which may be more restrictive than EU standards, and allows to take into consideration the individual characteristics of the receivers.

#### Requirements for treated wastewater quality in Sweden

In Sweden, to prevent eutrophication of lakes, the removal of phosphorus from municipal wastewater began in the 1970s (Mundt 2005). Currently, all water bodies in Sweden are defined as sensitive to eutrophication, which results in a unique tightening of regulations regarding the quality of wastewater discharged from WWTP.

Identifying phosphorus as the main limiting factor, conditioning the growth of aquatic vegetation, the Swedish legislation specifies the limit of TP in wastewater discharged into receivers at the level not exceeding 0.5 mg/l (Bjurhall 2004). Also, the 7-day biochemical oxygen demand (BOD_7_) limit value is more restrictive as compared to the rest of the EU Member States standards and is set at 15 mg/l (Kallqvist et al. 2002) (about 12.5 mg/l of BOD_5_; Öberg 2004). Regulations regarding the concentration of TN do not differ from those established by the UWWTD. The WWTPs in Sweden achieve a high level of phosphorus elimination mainly due to the widely used in Scandinavian countries the method of biological treatment intensified by additional chemical precipitation (Krizanac 2005).

#### Wastewater discharge regulations in Denmark

The Danish legal regulations regarding wastewater discharges are one of the most restrictive in the EU countries (Brix and Arias 2005; Christiansen and Kardel 2005). The implementation of eutrophication prevention policies was pioneered by Denmark since the first discharge limits concerning municipal wastewater were established by national law in the 1960s (Klinglmair et al. 2015). After implementing the UWWTD, Denmark has made another step toward nutrient pollution prevention by developing more strict discharge limits (Valero et al. 2018). Besides the pollutants permissible concentrations, the regulations include also the recommendations on wastewater treatment technologies (Vind 2017).

To support the nutrient reducing actions, Denmark has introduced a discharge tax concerning BOD_5_, TN, and TP. By this tax, the “Polluter Pays Principle” has been fully adapted and obligatory for WWTPs operators. The tax rates regarding the treated wastewater discharged into receiving waters are set for three parameters: BOD_5_ (2.47 Euro/kg), TN (4.44 Euro/kg), and TP (24.46 Euro/kg) using the Euro to Danish Krone exchange rate at 7.47.

#### Recommendations of the Helsinki Commission (HELCOM)

Along with the limits set by the UWWTD, stricter discharge limits were set by HELCOM (2007) in the Recommendations of the Baltic Marine Environment Protection Commission 28E/5, basing on the agreement of the Baltic Region countries Ministers of Environment in 2007.

According to HELCOM recommendations, WWTPs located in the Baltic Sea catchment are obliged to comply not only with the national legal regulations but also HELCOM requirements, which have set a minimum degree of reduction and allowable values for three basic indicators: BOD_5_, TN, and TP (HELCOM 2007). The requirements of HELCOM, as well as EU requirements, have been developed taking into account the PE value and are constantly undergoing an amendment toward an even greater reduction of pollutant loads discharged from the treatment plants, especially the loads of nutrients (Iho et al. 2015; Jetoo 2018).

### Conditions of Wastewater Discharge into the Receivers in Non-EU Countries

The states not belonging to the EU are often characterized by different approaches to establishing the legal regulations regarding the wastewater discharge into surface waters. A substantially different approach, for example, exists in the countries that were formerly part of the Soviet Union. The methodology of determining the conditions of wastewater discharge into receivers of various categories is based on the assumption, that the level of its treatment should ensure the normative water quality in the control cross-sections of individual water bodies (Neverova-Dziopak 2018). The maximum allowable load discharged from each WWTP is determined, taking into account the type and the specific characteristics of the receiver, the category of its use, and the construction of the wastewater outlet.

The degree of wastewater treatment is determined to take into account the degree of wastewater mixing with the receiving water and its background quality (Ministry of Natural Resources 1991, 1999). The above approach is still used, among others in Russia, Moldova, Kazakhstan, Uzbekistan, and other countries of the former Soviet Union (Buijs 2007, 2009; OECD 2011). Increasingly due to the high pollution degree of the aquatic environment, the quality standards set for surface waters being the wastewater receivers are often related directly to wastewater introduced into the receivers, i.e., the degree of wastewater dilution with the receiver water is not taken into account in this case. An overview of the analyzed legal requirements from non-EU countries and regions for regulating the conditions of wastewater discharges is presented in Tables 2 and 3.

**Table 2:** Legal requirements set for receiving surface water quality in Russia

Country	Water category	COD, mg/l	BOD, mg/l	NH_4_^+^–N, mg/l	NO_2_^−^–N, NO_3_^−^–N, mg/l	TN, mg/l	PO_4_^3−^–P, mg/l	TP, mg/l	References
Russia	Industrial fishing areas	n/n^a^	3.0^b^ (BOD_20_)	0.39	0.02 (NO_2_^−^–N) 9.1 (NO_3_^−^–N)	n/n	2.0 (0.2 in eutrophic waters, 0.15 in mesotrophic waters, 0.05 in oligotrophic waters)	n/n	Ministry of Natural Resources (1991, 1999), Gogina (2010)
	Source of water supply	15	3.0 (BOD_20_)	n/n	n/n	n/n	n/n	n/n	
	Recreation and water sports	30	6.0 (BOD_20_)	n/n	n/n	n/n	n/n	n/n	

^a^*n/n* not normalized parameter

^b^1.0 mg/l BOD_20_ = ~0.68 mg/l BOD_5_

**Table 3:** Legal requirements concerning treated wastewater discharges in non-EU countries/regions

Country/region	WWTP category	COD, mg/l	BOD_5_, mg/l	NH_4_^+^–N, NH_3_–N, mg/l	NO_2_^−^–N, NO_3_^−^–N, mg/l	TN, mg/l	PO_4_^3−^–P, mg/l	TP, mg/l	References
Belarus	<500 PE	125	35	n/n^a^	n/n	n/n	n/n	n/n	Ministry of Environment (2012)
	501–2000 PE	120	30	20	n/n	n/n	n/n	n/n	
	2001–10,000 PE	100	25	15	n/n	n/n	n/n	n/n	
	10,001–100,000 PE	80	20	n/n	n/n	20	n/n	4.5	
	>100,000 PE	70	15	n/n	n/n	15	n/n	2	
Switzerland	200–10,000 PE	60	20	2 (sum of NH_3_–N and NH_4_–N)	0.3 (NO_2_^−^–N)	n/n	0.8	n/n	The Swiss Federal Council (1998)
	>10,000 PE	45	15	2 (sum of NH_3_–N and NH_4_–N)	0.3 (NO_2_^−^–N)	n/n	0.8	n/n	
China (Taihu Lake catchment)	n/n	50	n/n	8 (NH_4_^+^–N, 5 in winter season)	n/n	15	n/n	0.5	Li et al. (2012)
USA	n/n	n/n	30	n/n	n/n	3–5 (areas sensitive to eutrophication)	n/n	1.0–0.1 (areas sensitive to eutrophication)	Sedlak (1991), US EPA (2012)
BC, Canada	Streams, rivers and estuaries	n/n	45 (10 if dilution ratio < 40:1)	n/n	n/n	n/n	0.5 (MDF^b^ > 50 m^3^/d)	1.0 (MDF > 50 m^3^/d)	British Columbia Office of Legislative Counsel Ministry of Attorney General (2005), US EPA (2012)
	Lakes	n/n	45	n/n	n/n	n/n	0.5 (MDF > 50 m^3^/d)	1.0 (MDF > 50 m^3^/d)	
	Open marine water	n/n	130 (MDF > 10 m^3^/d)	n/n	n/n	n/n	n/n	n/n	
	Coastal waters	n/n	45 (MDF > 10 m^3^/d)	n/n	n/n	n/n	n/n	n/n	
Dubai	Harbor area	100	50	2 (NH_4_^+^–N)	40 (NO_3_^−^–N)	10 (TKN^c^)	2	n/n	Government of Dubai (2010, 2018)
	Open Sea	n/n	30	5 (NH_3_–N)	n/n	n/n	0.1	n/n	

^a^*n/n* not normalized parameter

^b^*MDF* maximum daily flow

^c^TKN as a sum of organic nitrogen (N_org_) and NH_4_^+^–N

#### Methodology for determining the permissible concentrations of pollutants in treated wastewater in Russia

The current wastewater standards regulations in Russia provide a set of standardized indicators and determine their permissible values in surface waters for the following parameters: BOD, COD, total suspended solids (TSS), NH_4_^+^–N, nitrites (NO_2_^−^–N), nitrates (NO_3_^−^–N), and orthophosphates (PO_4_^3−^–P) (Stefanova et al. 2019). Surface water bodies located in the Russian part of the Baltic Sea basin have been included in the areas of industrial fisheries, for which the most stringent standards are applied (Nikolajew et al. 2008). Therefore, maximum concentrations of pollutants in treated wastewater are set at a maximum permissible level of pollutants in surface waters used as fishery areas.

The standards for surface water quality in Russia are established depending on the category of their use and they are the basis for establishing the legal rules of wastewater discharge into surface waters. According to this approach, the quality of wastewater discharged into the receiver should be determined in such a way that after introducing the parameters of the receiver’s water quality do not exceed the set limits presented in Table 2 depending on the type of water use. Moreover, this approach allows in each specific case to adjust the quality of treated wastewater to the condition in the receiver and its self-purification capacity.

Since northwestern regions of Russia with extensive urban-industrial agglomerations are located in the Baltic Sea catchment area (Sankt Petersburg and Kaliningrad), the limits for WWTPs discharging their effluents to the Baltic Sea are set according to HELCOM recommendations.

#### Quality standards for treated wastewater in Belarus

The objectives and basic principles of standardization in the field of water protection in Belarus are contained in the Technical Code under the title: “The order of establishing the norms of permissible discharges of chemical substances and other pollutants in wastewater composition” (Ministry of Environment 2012). Permissible concentrations of pollutants are set for COD, BOD_5_, TSS, NH_4_^+^–N, TN, and TP content depending on PE, determined based on the unit BOD_5_ load.

The permissible content of other pollutants in wastewater discharged into water receivers and the required level of their reduction are determined taking into account the intensity of wastewater outflow, the concentration of pollutants in the receiver and its assimilation capacity following the guidelines enclosed in the Technical Code (Ministry of Natural Resources 1991, 1999; Gogina 2010).

#### Effluent quality standards in Switzerland

Water governance in Switzerland is divided into three levels: federal, cantonal, and municipal (Federal Ministry of Environment Nature Conservation and Nuclear Safety 2002). The main legal framework governing water resources in Switzerland is the Federal Water Protection Law (WPL) defined at the federal level. Based on the WPL the Waters Protection Ordinance (WPO) was adopted on October 28, 1998 by the Swiss Federal Council and is still in force (Bucheli et al. 2010).

Switzerland as a non-EU country has its national legal regulations concerning water and wastewater management which mainly corresponds with EU water policy (Eggen et al. 2014). Besides the Swiss efforts for maintaining the waters quality at the national level, Switzerland fulfills its international responsibilities by active participation in international commissions such as the International Commission for the Protection of the Rhine, the International Commission for the Protection of Lake Constance, the Commission for the Protection of the Waters of Lake Geneva, the International Commission for the Protection of Italian-Swiss Waters, and the Commission for the Protection of the Marine Environment of the North-East Atlantic (Lieberherr 2011).

The Swiss legal requirements concerning municipal wastewater discharge are developed for basic parameters such as BOD_5_, COD, and TSS concerning also three main nutrients: ammonium, nitrites, and orthophosphates. However, the requirements for ammonium content are applied when it is potentially detrimental to the water quality of the water body, and if the wastewater temperature is higher than 10 °C. Additional requirements concerning orthophosphates discharged into sensitive waters apply in the lakes catchments, on watercourses beyond the lakes, and for WWTPs above 10,000 PE, situated on watercourses in the catchment area of the Rhine downstream of lakes (The Swiss Federal Council 1998). Dissolved organic carbon is also limited for effluents above 2000 PE. According to the WPO, the discharge concentrations and the removal efficiency are considered together, in contrast to EU standards where both criteria can be interchangeable.

Switzerland has achieved great success in reducing phosphorus loads (Brunner et al. 2019). The phosphorus concentrations in Swiss lakes have steadily declined since the 1980s (Rodríguez-Murillo et al. 2015) and the current state of Swiss lakes can be generally described as good (Tu et al. 2019). Unfortunately, due to the soil enrichment in phosphorus compounds on agricultural lands of high livestock density within the lake catchment areas, a further water quality improvement cannot be guaranteed for all lakes (Bucheli et al. 2010; Ferré et al. 2019). To provide the highest possible protection of the lakes susceptible to eutrophication the effluent quality requirements should be stricter for each wastewater receiver. For example, for wastewater discharged into Lake Lugano, the TP allowable concentration is set at the level of 0.3 mg/l (OECD 2007). So in the terms of eutrophication, the Swiss effluent standards take into account the sensitivity of individual receivers to eutrophication, treating phosphorus as a key factor.

#### Quality standards for treated wastewater in China

One of the characteristic features of surface waters state in China is the wide variation of water pollution levels in different geographic regions of the country. In general, the level of surface water pollution is relatively low, but in the Pearl and Yangtze river basins, one of the highest levels of water pollution is observed (Guo 2007).

The reason for the poor state of the water environment is the intensive industrial activity and a high degree of urbanization in the region (Yue et al. 2018). The worst situation associated with water eutrophication is observed in Lake Taihu located about 100 km west of Shanghai in the Yangtze River delta, which for centuries has served as a natural retention reservoir for irrigation of rice fields, fishing, and shipping. In 2007, the waters of the lake were classified into the lowest quality category (category V+) (Li et al. 2012).

To prevent a deteriorating of this situation, in 2008 the Ministry of the Environment Protection of China has established the special limits for wastewater discharges into water receivers in ecologically sensitive areas. New standards for the 11 quality parameters for industrial wastewater in the Yangtze basin were also developed (Wang and Wang 2009).

In the same year, the most stringent local standards for municipal wastewater discharge were established for the Lake Taihu catchment area, which covered the following four parameters: COD, NH_4_^+^–N, TN, and TP (Liu et al. 2013).

The low permissible concentration of phosphorus in treated wastewater allows concluding that also in China the key role of phosphorus in the development of eutrophication processes in inland waters is noticed. The permissible TP concentration at 0.5 mg/l is even more stringent than the standards applicable in most European countries. However, this value is a regional standard and is applied only in a specific catchment. In contrast, in the rest of China’s water areas, the permissible concentration of TP is established at the level of 1.0 mg/l and TN at 15 mg/l (Li et al. 2012).

The Chinese guidelines also require the limiting of NH_4_^+^–N content to a maximum of 8.0 mg/l (5.0 mg/l in winter season), which is considered to be one of the most eutrophic forms of nitrogen compounds (Zhang et al. 2015). As indicated by the experience from WWTPs in China, actually the municipal wastewaters are characterized by an unfavorably low ratio of COD to nitrogen (C/N), which causes a problem with biological denitrification, so the removal of TN is a great challenge (Bodik et al. 2009).

The methodology of determining the conditions for wastewater discharge into receivers, taking into account their regional characteristics, seems to be justified in such large and geographically and climatically diversified territories like China. A similar regional approach to regulations regarding the quality of wastewater discharged into receivers is also applied in the countries of North America—USA and Canadian provinces.

#### Quality standards for treated wastewater in the United States of America

Legal regulations regarding the wastewater discharge into surface waters in the United States are determined based on the water state of the receiver and its sensitivity to eutrophication (Carey and Migliaccio 2009). The USA, like China, has a wide territory with a large diversity of geographic, climatic conditions and the degree of urbanization. Unlike in China, the permissible values obligatory for the entire USA area are set only for BOD_5_ and TSS: for both parameters, the maximum permissible content in wastewater amounts 30 mg/l with a reduction of at least 85%, while the COD value in the national scale is not standardized (Sedlak 1991).

The permissible concentrations regarding the nutrients content in wastewater are set only for water bodies sensitive to eutrophication, e.g., Lakes Tahoe and Occoquan, Great Lakes, Chesapeake Bay, the northern part of the Gulf of Mexico, and others. In the USA, the quality standards of wastewater discharged into waters susceptible to eutrophication have a regional character (Li et al. 2012).

The conditions for wastewater discharge into water bodies exposed to eutrophication in the USA can be considered as one of the most stringent. The achievement of permissible effluent TP concentration of 0.1 mg/l (Sedlak 1991) is possible only when using exceptionally capital-intensive technologies, which are characterized by increased energy consumption and greenhouse gases emission, large amounts of chemical reagents, and high costs of processing and utilization of an increased amount of sewage sludge.

National guidelines applicable in the USA to treated wastewater discharged into the receivers resistant to eutrophication are much less restrictive. On the other hand, the lack of requirements regarding TN concentration standards makes it possible to avoid the use of expensive technological systems for advanced nitrogen removal and justifies the use of basic systems of biological treatment with chemical precipitation only when necessary. This approach seems to be justified from economic and ecological points of view.

#### Quality standards for treated wastewater in British Columbia (BC), Canada

In BC province, the quality of wastewater discharged into the receiver depends on the maximum daily wastewater flow rate from the treatment plant and the type of the receiver. Three WWTP capacity categories have been established by BC legislation: below 10 m^3^/d, 10–50 m^3^/d, and above 50 m^3^/d. Three types of receivers were also determined: (1) streams, rivers, and estuaries, (2) lakes, and (3) marine waters. The standard values for BOD and TSS were established with consideration of the properties of the receiver. The ratio of water receiver flow intensity to treated wastewater flow intensity is also taken into account: it allows to consider the degree of wastewater dilution. The 40:1 ratio was assumed as the limit value, while under the ratio below 10:1 the wastewater discharge is prohibited (British Columbia Office of Legislative Counsel Ministry of Attorney General 2005).

For the areas particularly sensitive to eutrophication the regulations should be more restrictive. For example, regarding the treated wastewater discharges to the Okanagan and Christina Lakes or the Thompson, Cowichan, Nicola, and Cheakamus rivers, the maximum permissible TP concentration in discharged wastewater is only 0.25 mg/l (US EPA 2017).

The conditions for wastewater discharge into surface wasters in BC do not include the obligatory requirements for effluent nitrogen content. Such requirement is applied in the case of chosen water bodies (Sedlak 1991). For example, for municipal and industrial wastewater discharged into Okanagan Lake the maximum concentration of TN is 6.0 mg/l (British Columbia Office of Legislative Counsel Ministry of Attorney General 2005). A methodological approach to standardizing the quality of wastewater discharged to receivers in Canada can also be referred to the regional approach considering the individual characteristics of different receivers.

#### Emirate of Dubai

Wastewater management in Dubai is a matter of special importance due to the lack of freshwater resources. The task of wastewater management is not only marine water protection but also the production and accumulation of additional water resources for the needs of residents and irrigation. The rapid population growth in the United Arab Emirates (UAE) along with intensive consumption of drinking water has put heavy pressure on the limited national water resources. Recycled treated wastewater is a valuable source of potable water that can be used as an alternative to marine water desalination (Khan and Dghaim 2016). The authorities of UAE aware of the validity of water issue importance and its influence on national income level from tourism, reveled in September 2017 the UAE Water Security Strategy 2036. This document aims to ensure sustainable access to water in line with standards of the World Health Organization and the UAE’s vision of sustainability and prosperity (UAE’s Ministry of Energy & Industry 2017).

The Dubai Emirate Government implemented the Water Environment Regulation EN-5.0 (Government of Dubai 2018) which defined the quality limits of wastewater discharge. Dubai’s legislation on treated wastewater discharge established the effluent quality standards depending on the type of two main receivers: the Dubai Harbor area and the open waters of the Persian Gulf. The effluent standards for wastewater discharge to the Dubai Harbor concern the content of NH_4_^+^–N, BOD_5_, COD, nitrate nitrogen (NO_3_^−^–N), total Kjeldahl nitrogen, and PO_4_^3^^−^–P, while discharges limits to the open waters of the Persian Gulf concern the content of NH_3_–N, BOD_5_, and PO_4_^3−^–P (Government of Dubai 2010, 2018).

These standards have been established to minimize the negative impact on the harbor and the Persian Gulf water quality, the Gulf ecosystem, and the local fishing industry (Government of Dubai 2018). The standards take into account not only total but also mineral forms of nutrients, while for more polluted port waters they are less restrictive.

### Analysis of Different Approaches to Wastewater Quality Standardization

The analysis of the existing approaches to developing of requirements for treated wastewater quality discharged into surface waters, with particular emphasis on the content of the biogenic compounds, confirms the complex nature of water quality standardization and setting the value of permissible concentration/loads of pollutants introduced into the aquatic environment together with municipal wastewaters.

The existing methodological approaches to establishing the quality requirements for discharged wastewater in different countries are based on various assumptions which can be grouped as follows:

1. Permissible concentrations of pollutants (BOD, COD, TSS, TN, TP, etc.) in wastewater and/or the reduction efficiency rates established at national, regional or local levels, which should be obligatorily achieved in the treatment process.
2. Uniform quality standards for treated wastewater applicable throughout the country.
3. Environmental standards regarding the water quality in the receiver, which should not be deteriorated as a result of treated wastewater discharge.
4. Technological standards recommending the use of certain technological systems or processes, without specifying the allowable values of pollutants in treated wastewater.

**Fig. 3:**
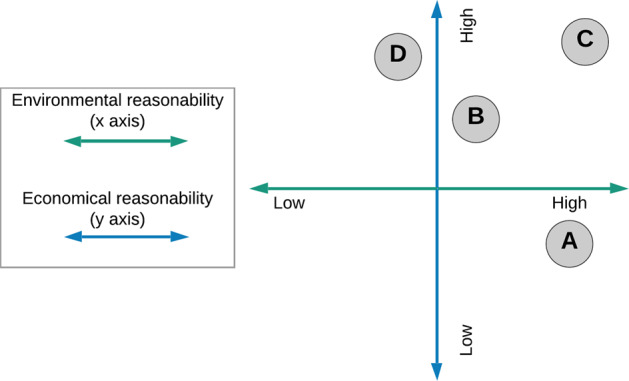
Wastewater discharges standards assessment matrix

The above approaches have been assessed in terms of economic and environmental reasonability. The assessment results are presented in Fig. 3.

Regarding the variety of methodological approaches, the overview of wastewater quality requirements is presented in Tables 1–3. The comparison of the analyzed national legal regulations (besides limits introduced by the UWWTD) in the EU Member States concerning treated wastewater effluent quality is presented in Table 1.

An example of a different approach to establishing the allowable content of wastewater pollutants discharged in surface waters is presented in Table 2. The established surface water quality standards are the basis for determining the allowable concentrations of pollutions in discharged wastewater. The level of wastewater treatment should ensure the normative water quality presented in Table 2 in the control cross-sections of the recipient. The approach is illustrated by the example of rules for the protection of surface waters from pollution by wastewater in Russia.

The set of legal requirements concerning treated wastewater discharges in selected non-EU countries/regions is shown in Table 3.

The analysis of the current requirements for various countries is presented in Tables 1–3 which have shown essential methodological differences in setting the effluent quality standards and determining the conditions of treated wastewater discharge into surface waters in the EU Member States and the non-EU countries from different regions of the world.

The EU Member States were obliged to implement the uniform quality standards for treated wastewater in the form of maximum allowable values of pollution indicators or the minimum level of their reduction established by European legal acts. It is currently the most common procedure, which is characterized by simplicity, easy formal implementation allowing controlling officially the compliance with standards. Unfortunately, the use of such an approach also has drawbacks. In some cases, it leads to the application of too strict requirements and unjustified high costs of wastewater treatment or, on the contrary, to the introduction of insufficiently treated wastewater to the receiver.

The current situation caused a tendency to move away from unitary water and wastewater quality standards, valid throughout the entire territory of different countries, for their regionalization and differentiation, taking into account the properties of separate water bodies. This trend was reflected in the dependence of the demanded wastewater treatment efficiency on the size of the agglomeration (PE value) and the intensity of wastewater effluent, on hydrodynamic and hydrological properties of receivers and their self-purification capacity or water susceptibility to eutrophication. In many EU countries local and regional standards for treated wastewater quality also have been developed in order to meet the assumptions of the EU Water Framework Directive, which introduced an ecosystem approach to water resources management and protection. In such way the ecological properties and ecosystem response of individual recipients can be considered in quality standards.

A common feature that characterizes recent legislation changes in different countries is the restriction of the quality requirements for treated municipal wastewaters in connection with the deterioration of surface water state. Due to the need to prevent the commonly occurring eutrophication, these changes concern mainly the content of biogenic compounds, which resulted in the development of permissible concentrations of TN and TP in treated wastewater, and only in some counties such standards were elaborated also for their mineral (bioavailable) forms.

Regarding the bioavailability context of various N and P compounds it is a well-known fact that predominantly dissolved inorganic (mineral) forms of nutrients are directly available to aquatic vegetation (Berge and Kallquist 1995; Gao et al. 2010; Gu et al. 2011), while organic forms are not readily available for plant hydrobionts (Granéli et al. 1990). Therefore, it is the share of these bioavailable nutrient forms that determine the eutrophication potential of wastewater discharged into receivers. Nitrates and ammonium are considered to be the most available N compounds (Nakajima et al. 2006; Håkansson and Bryhn 2008), while orthophosphates (H_2_PO_4_^−^, HPO_4_^2^^−^, or PO_4_^3−^) are the only directly available P form for planktonic algae and bacteria (Warwick et al. 2013; Venkiteshwaran et al. 2018). Therefore, it should be emphasized, that not only total nutrient forms but mainly their inorganic compounds should be limited by legal regulations aimed at mitigating eutrophication. The approach to eutrophication mitigation based only on TN and TP concentrations does not include the nutrients bioavailability context and results in less efficient protection against eutrophication.

It should be noted that in order to prevent eutrophication in the EU Member States the concentrations of nutrients are obligatory limited, but the limits are mainly imposed on TP and TN (more rarely, NH_4_^+^–N). However, in some countries (Dubai, USA, Canada, and selected countries of the former Soviet Union) the standards for bioavailable (inorganic) forms of nitrogen and phosphorus were elaborated with consideration of the main limiting factor in fresh and/or marine waters.

The analysis of the principles of wastewater discharge in Russia (which is still used in some countries that once belonged to the former Soviet Union) allowed distinguishing a fundamentally different approach based on environmental standards set for the receivers of various categories of water use. These standards are the basis for setting the limits of pollutant loads discharged into the receiver of a specific category. Such an approach is more complicated and requires the calculating of permissible concentration of each pollutant for each WWTP depending on the type of receiver, but it allows the consideration of the properties of a specific receiver and its assimilation capacity.

In the case of the non-EU countries discussed, new trends in the development of modern strategies for wastewater quality standards can also be observed, and two basic models for the development of water protection strategy can be distinguished.

The first model assumes a resignation from the previous methods of determining the treated wastewater quality standards and switching to a mixed system. In practice, this means, that in the case of municipal wastewater the quality standards are based on EU regulations setting the maximum permissible concentrations of pollutants or minimum levels of their reduction. Whereas in the case of industrial wastewater discharge the quality standards are set taking into account the best available technologies.

The second model assumes the modification of the existing system of environmental standards with the elimination of its disadvantages and the implementation of some assumptions of the EU regulations, such as reducing the number of standardized indicators and introducing the less restrictive standards for some indicators or vice versa, their tightening (Ministry of Natural Resources 1999).

A characteristic feature of the methodology for determining the effluent standards in Switzerland, USA, Canada, and China is the application of less restrictive national norms and very strict regional standards for sensitive wastewater receivers potentially endangered by eutrophication. This approach allows reducing the discharged loads of nitrogen or phosphorus with the consideration of the knowledge about the receiver properties and its self-purification capacity. This approach seems to represent a reasonable compromise between the ecological and economic aspects of wastewater treatment.

### Recommended Legislation Policy Directions

By analyzing various eutrophication mitigation approaches used in different countries the following recommendations are suggested to consider while establishing eutrophication aimed strategy:

1. The main criterion for determining the conditions of wastewater discharge to the receivers should be the permissible loads of individual pollutants, taking into account other nutrient sources of their delivering into the receiver such as agriculture, natural background, storm water, atmospheric deposition, etc.
2. The total load of individual polluting substances discharged from different sources to the receivers should not exceed their assimilation capacity.
3. The permissible concentrations of pollutants in discharged wastewater and their permissible loads should be regionalized according to the specific conditions in the receiving water body, its type, and the receiver ecosystem response to individual pollutants loads.
4. When developing legal requirements for the nutrients discharge to the receivers, it is necessary to determine the permissible values of their bioavailable forms, which condition the eutrophication potential of wastewater and constitute the main factor of eutrophication intensification.
5. The economic balance between wastewater treatment costs and economic losses due to ecological damage when introducing insufficiently treated wastewater to receiving waters should be maintained. The unjustified use of very expensive wastewater treatment technologies contradicts the principles of sustainable development, claiming to ensure a balance between economic and ecological aspects of development. In addition, when the complexity degree of the wastewater treatment technologies increases, the adverse effects on other elements of the environment are also increased (greenhouse gases emissions, energy consumption, sewage sludge amount, etc.).

## Conclusions

During the last decades, the implemented legal regulations concerning treated wastewater discharge standards did not lead to a satisfying effect on the improvement of surface water state. In many cases, the main barriers in restoring and maintaining good water ecosystems state are the uncertainties about the possible socioeconomic effects of environmental regulations. So far, the factors and mechanisms of one of the fundamental processes conditioning the surface water state—eutrophication process—were not sufficiently taken into account by existing regulations that resulted in serious financial expenditures without a visible effect.

The results of the analysis of methodological approaches to wastewater quality standardization in different countries and regions allow stating that the receiver-oriented policies show the most promising results (e.g., Switzerland, North America, and China). Unfortunately, many interregional regulations (e.g., European UWWTD) do not follow this path establishing the unified wastewater standards. Moreover, the climate differences, seasonality factors, type of recipient, the bioavailability of nutrients seem to be missing in the EU legislation for the Member States.

In the light of the above considerations, there is still an urgent need for further research of eutrophication development factors, especially in sensitive water bodies, including the understanding of nutrient bioavailability aspects, the key limiting factors, the possible reaction of water ecosystem for specific pollutant loads, nutrients ratios, climate influence, etc. The effluent standards should take into account the response of the ecosystem to pollution loads, its assimilation (self-purification) capacity. The standards must be ecologically justified and ensure the ecological safety of receivers. Such standards cannot be introduced without economical reasonability and do not lead to an overstatement of local budgets and the willingness of residents to bear the environmental costs.
